# Supplementary material for: Computational Selectivity Assessment of Protease Inhibitors against SARS-CoV-2
Source: Int J Mol Sci. 2021 Feb 19;22(4):2065. doi: 10.3390/ijms22042065 (PMC7922391; doi:10.3390/ijms22042065)
Supplement: Supplementary file 1 [file ijms-22-02065-s001.pdf]

Supporting Information: Computational Selectivity Assessment of Protease Inhbitiors against SARS-CoV-2

André Fischer,[†,¶] Manuel Sellner,[†,¶] Karolina Mitusińska,[‡,¶] Maria Bzówka,[‡,¶] Markus A. Lill,[†] Artur Góra,[*,‡] and Martin Smieško[*,†]

†*Computational Pharmacy, Department of Pharmaceutical Sciences, University of Basel, Klingelbergstrasse 61, 4056 Basel, Switzerland*

‡*Tunneling Group, Biotechnology Centre, ul. Krzywoustego 8, Silesian University of Technology, 44-100 Gliwice, Poland*

¶*These authors contributed equally to this work*

E-mail: a.gora@tunnelinggroup.pl; martin.smiesko@unibas.ch

Abstract

The pandemic of the severe acute respiratory syndrome coronavirus 2 (SARS-CoV-2) poses a serious global health threat. Since no specific therapeutics are available, researchers around the world screened compounds to inhibit various molecular targets of SARS-CoV-2 including its main protease (M^{pro}) essential for viral replication. Due to the high urgency of these discovery efforts, off-target binding, which is one of the major reasons for drug-induced toxicity and safety-related drug attrition, was neglected. Here, we used molecular docking, toxicity profiling, and multiple molecular dynamics (MD) protocols to assess the selectivity of 33 reported non-covalent inhibitors of SARS-CoV-2 M^{pro} against eight proteases and 16 anti-targets. The panel of proteases

included SARS-CoV M^{pro}, cathepsin G, caspase-3, ubiquitin carboxy-terminal hydrolase L1 (UCHL1), thrombin, factor Xa, chymase, and prostasin. Several of the assessed compounds presented considerable off-target binding towards the panel of proteases, as well as the selected anti-targets. Our results further suggest a high risk of off-target binding to chymase and cathepsin G. Thus, in future discovery projects, experimental selectivity assessment should be directed toward these proteases. A systematic selectivity assessment of SARS-CoV-2 M^{pro} inhibitors, as we report it, was not previously conducted.

Supporting Results and Discussion

Sequence and active site comparison

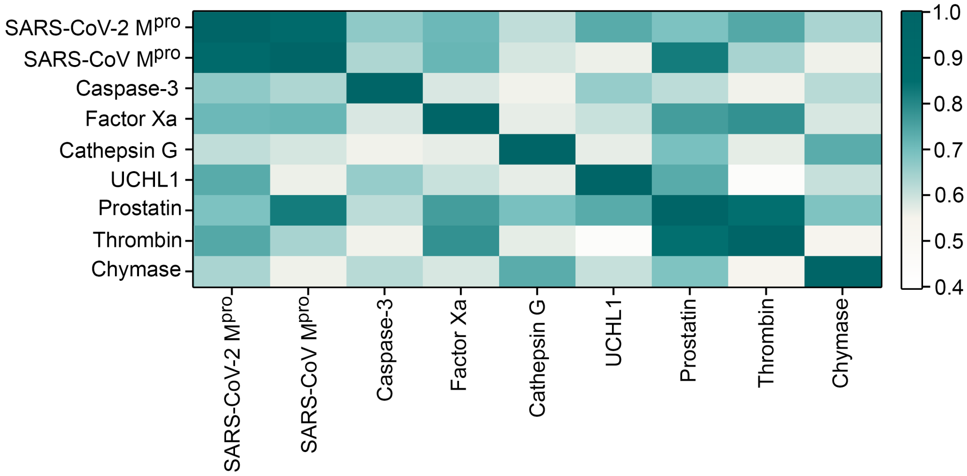

Figure S1: Active site similarity among all considered proteins determined by FuzCav.

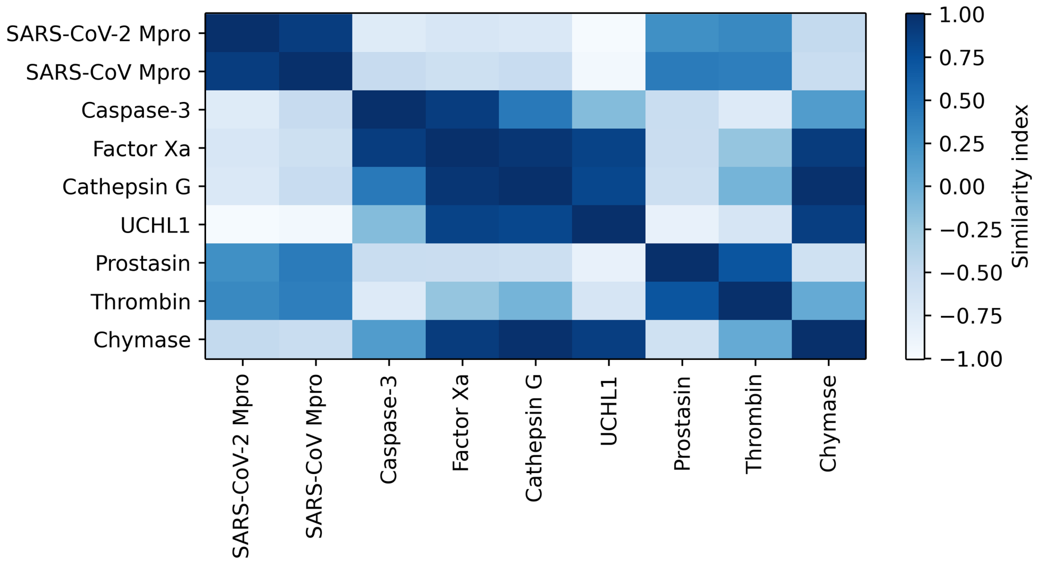

Figure S2: Hodgkin similarity index between binding cavities of all considered proteins determined by PIPSA.

Table S1: Comparison of geometrical parameters of the off-targets considered in this study.

**Protein**	**MAV** [Å^3][a]
SARS-CoV-2 M^{pro}	175 ± 46
SARS-CoV M^{pro}	352 ± 129
Caspase-3	340 ± 68
Factor Xa	379 ± 98
Cathepsin G	225 ± 102
UCHL1	918 ± 250
Prostasin	770 ± 97
Thrombin	842 ± 141
Chymase	328 ± 90

[a] Maximal available volume of the proteases' active sites.

Protease selectivity assessed by molecular docking

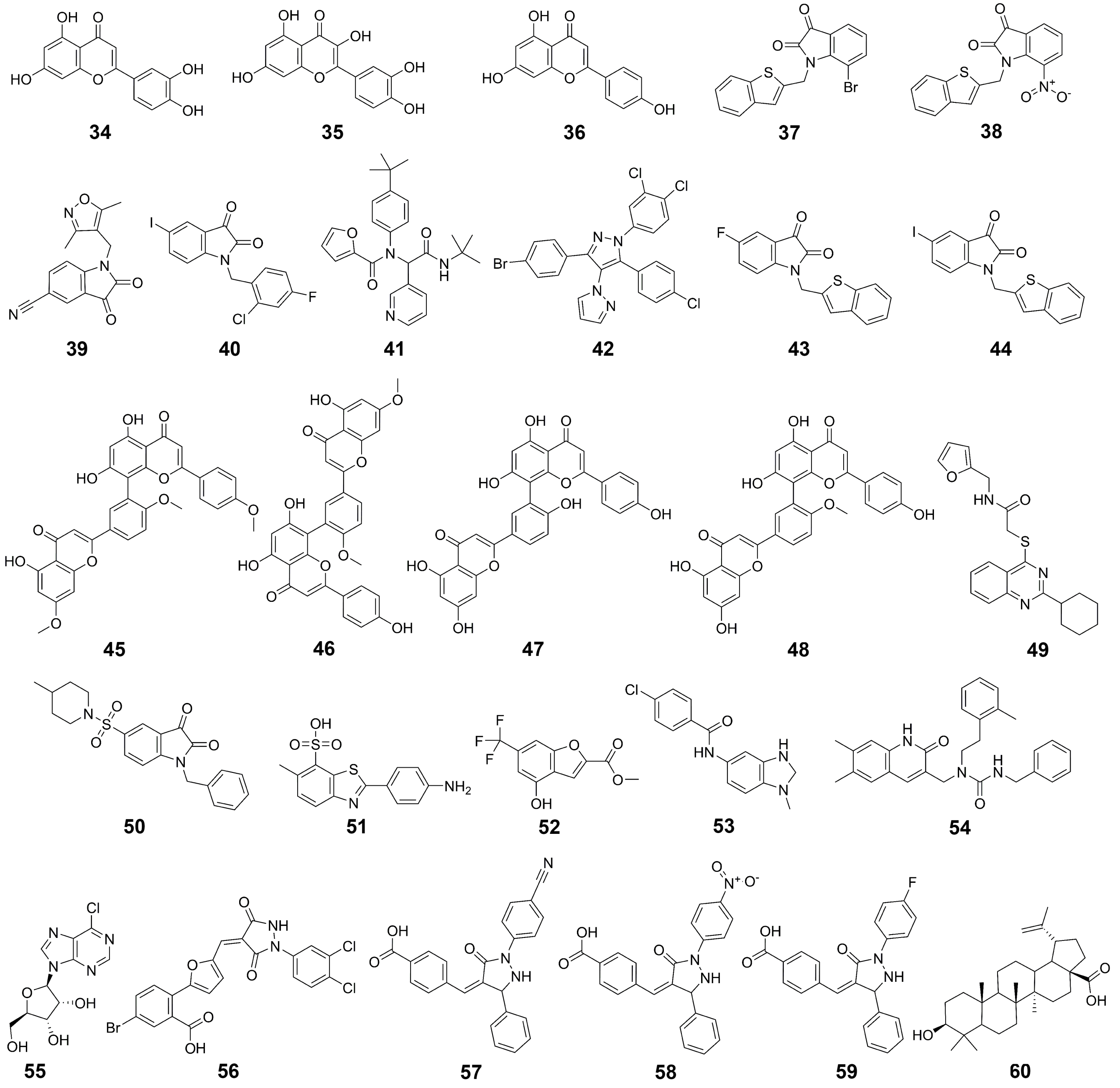

Figure S3: Selected actives of SARS-CoV M^{pro}.

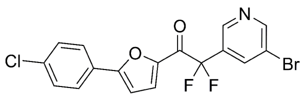

61

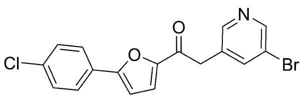

62

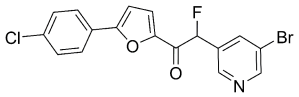

63

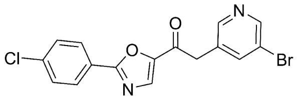

64

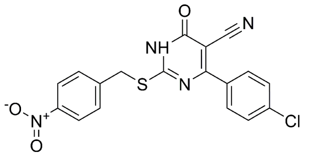

65

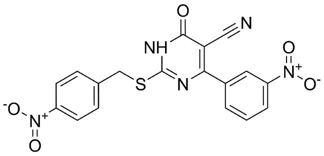

66

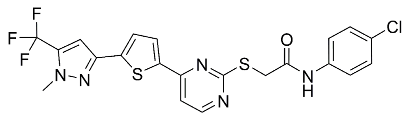

67

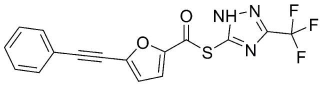

68

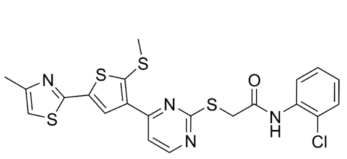

69

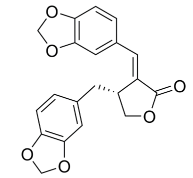

70

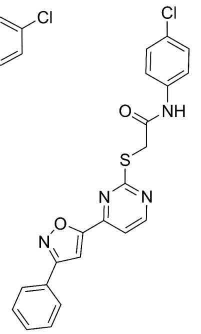

71

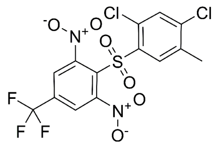

72

Figure S4: Selected actives of SARS-CoV M^{pro} (continued).

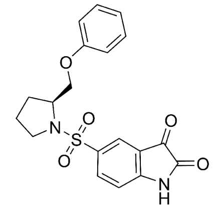
73

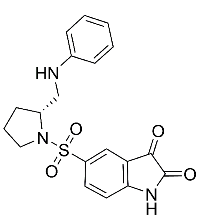
74

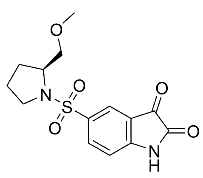
75

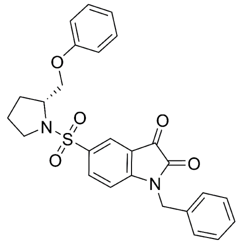
76

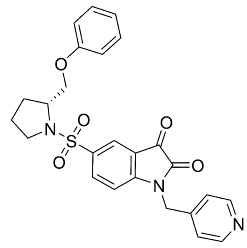
77

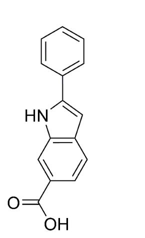
78

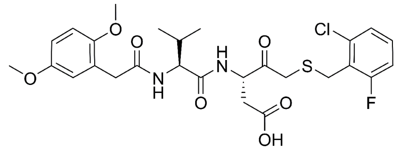
79

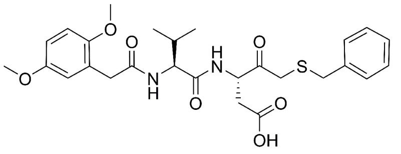
80

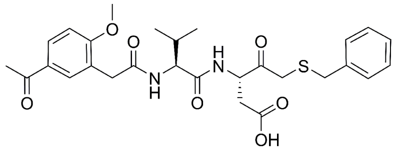
81

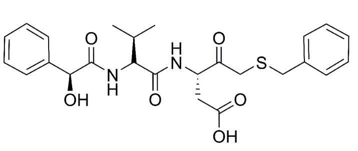
82

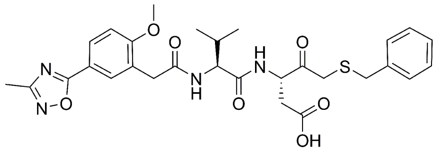
83

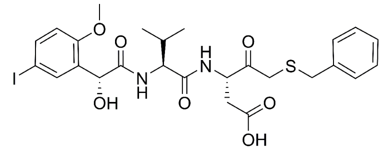
84

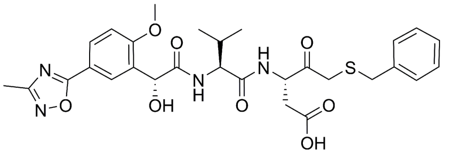
85

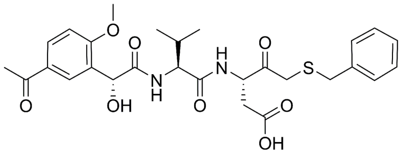
86

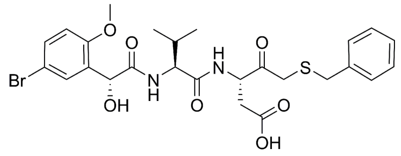
87

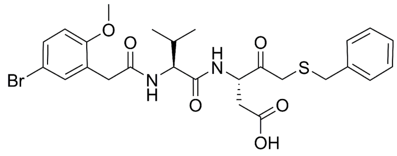
88

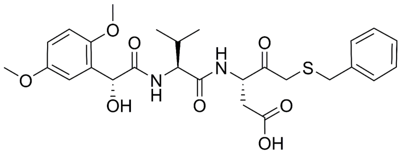
89

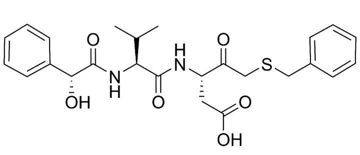
90

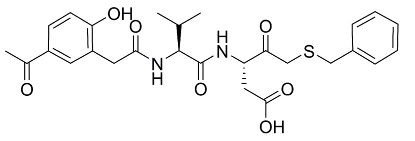
91

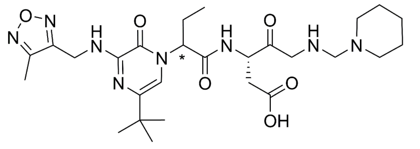
92

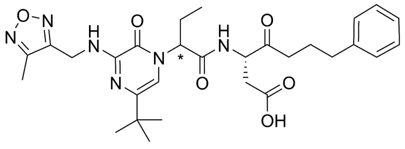
93

Figure S5: Selected actives of caspase-3.

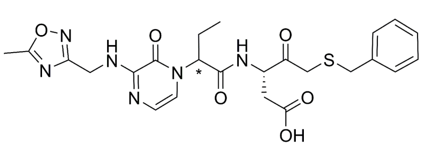

94

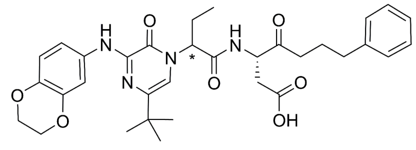

95

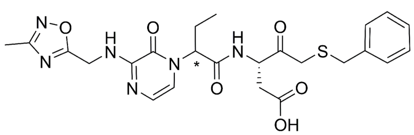

96

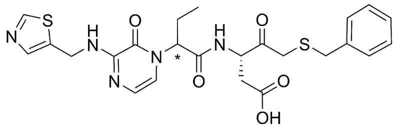

97

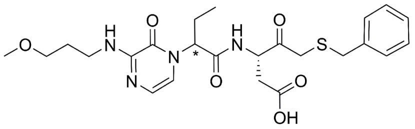

98

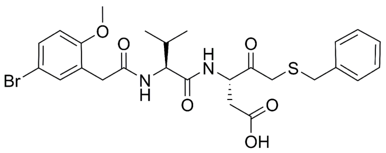

99

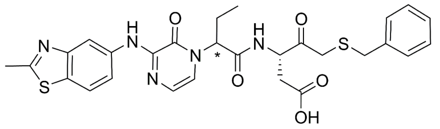

100

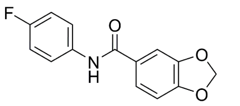

101

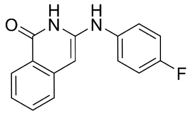

102

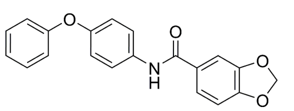

103

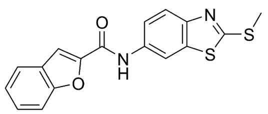

104

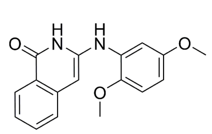

105

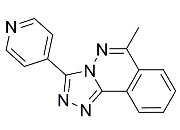

106

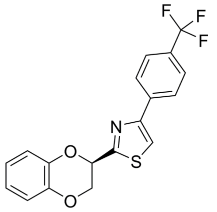

107

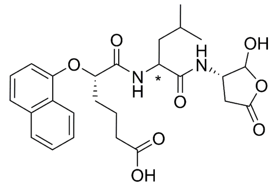

108

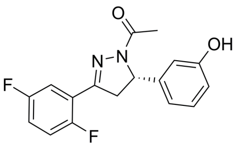

109

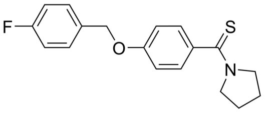

110

Figure S6: Selected actives of caspase-3 (continued).

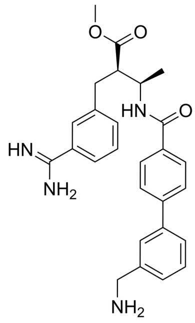

111

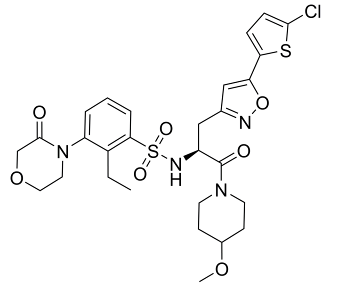

112

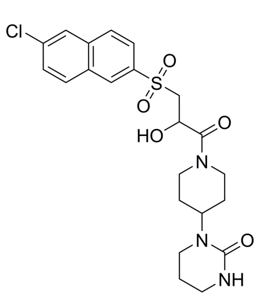

113

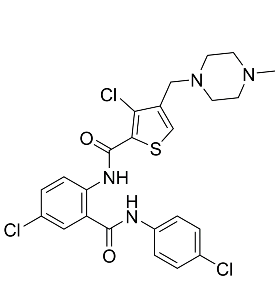

114

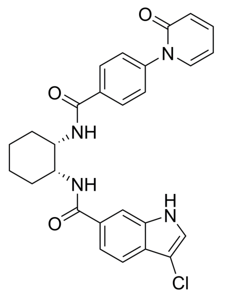

115

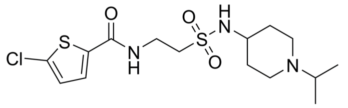

116

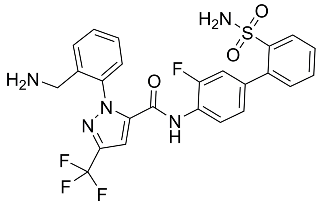

117

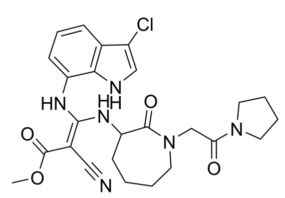

118

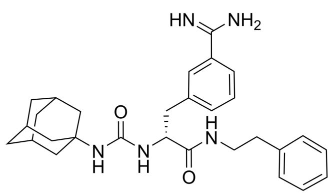

119

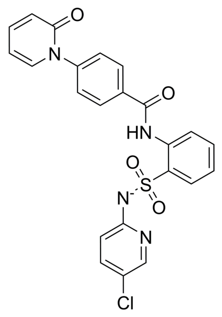

120

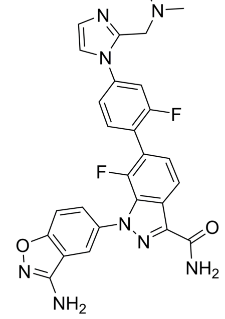

121

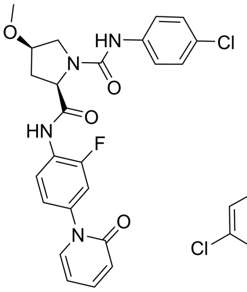

122

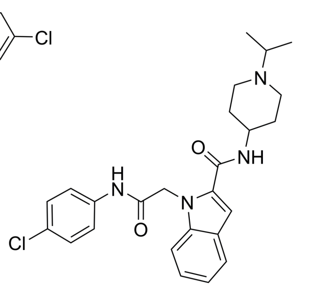

123

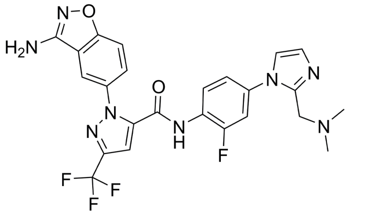

124

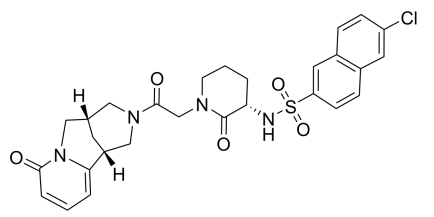

125

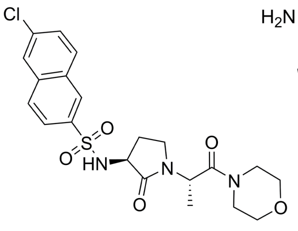

126

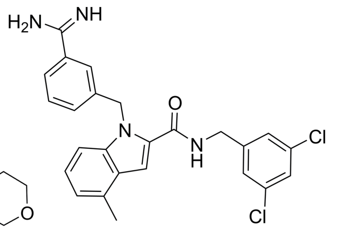

127

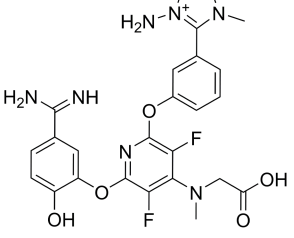

128

Figure S7: Selected actives of factor Xa.

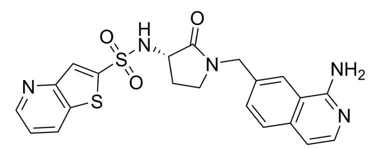

129

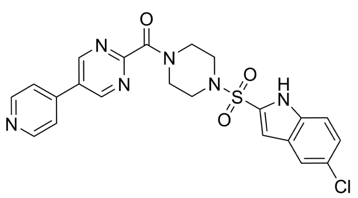

130

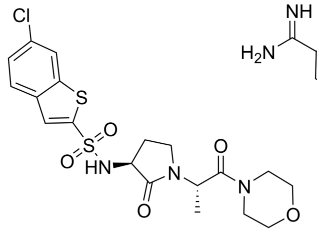

131

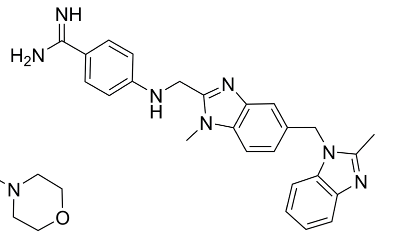

132

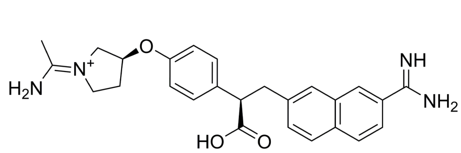

133

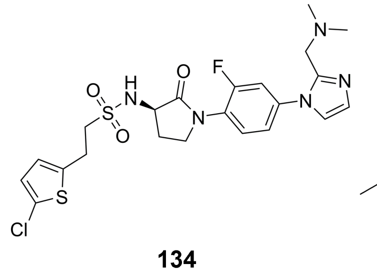

134

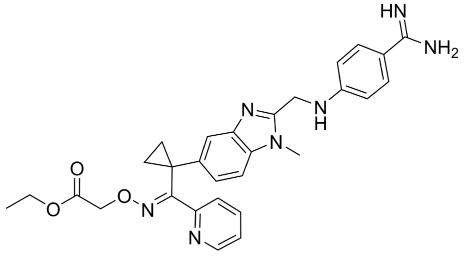

135

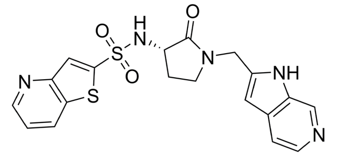

136

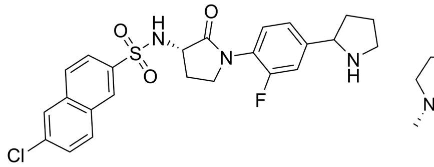

137

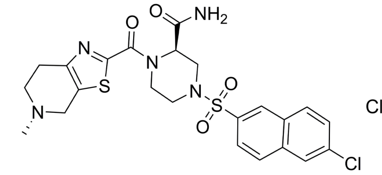

138

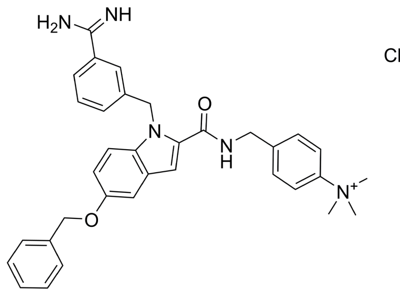

139

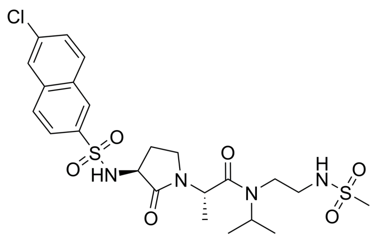

140

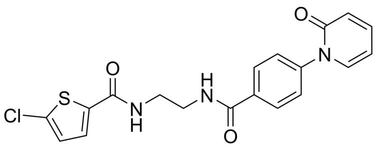

141

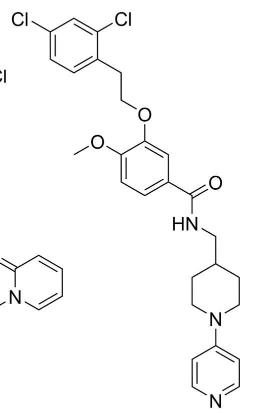

142

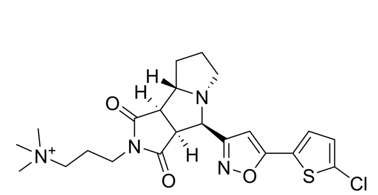

143

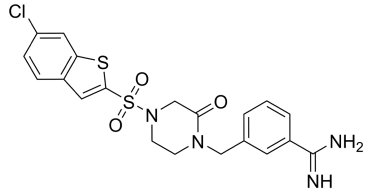

144

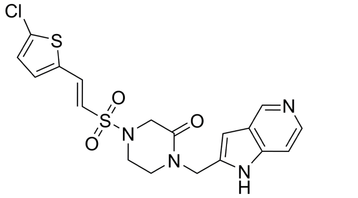

145

Figure S8: Selected actives of factor Xa (continued).

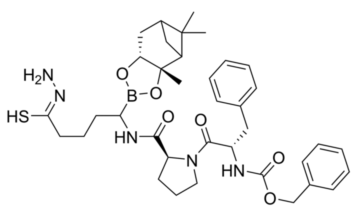
146

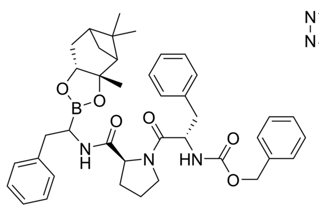
147

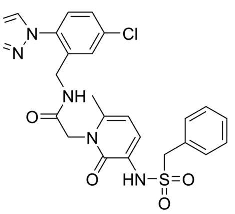
148

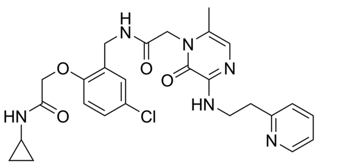
149

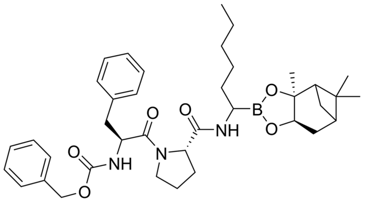
150

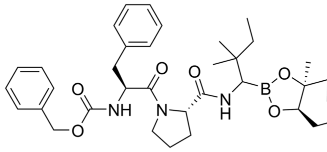
151

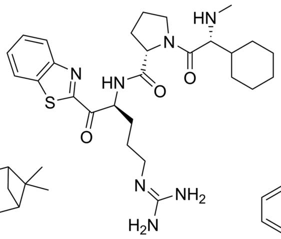
152

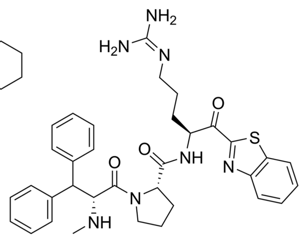
153

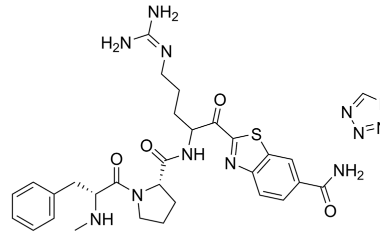
154

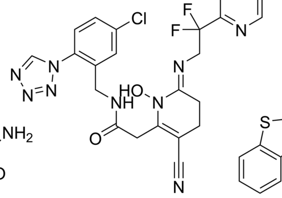
155

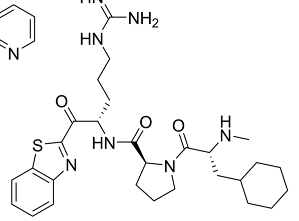
156

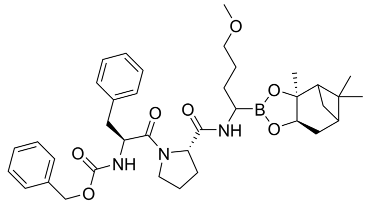
157

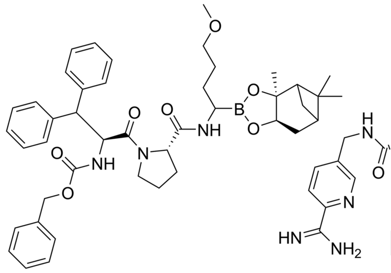
158

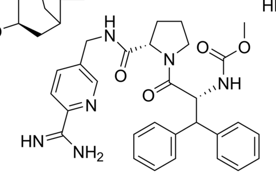
159

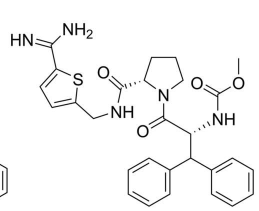
160

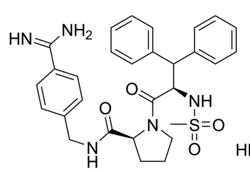
161

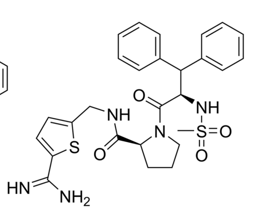
162

Figure S9: Selected actives of thrombin.

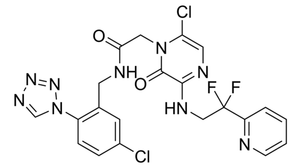
162

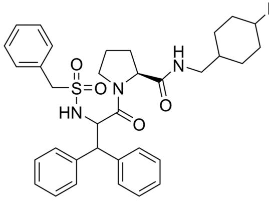
163

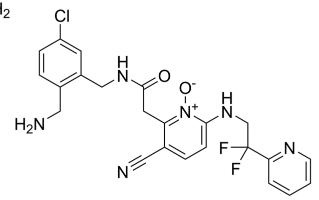
164

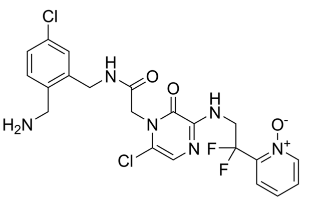
165

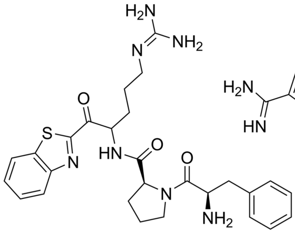
166

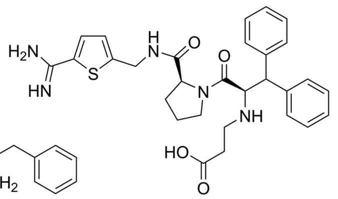
167

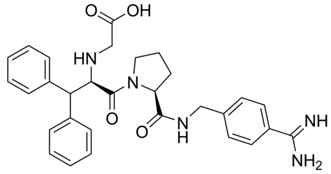
168

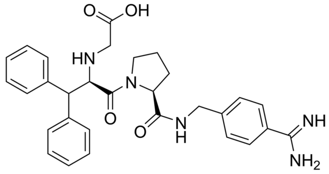
169

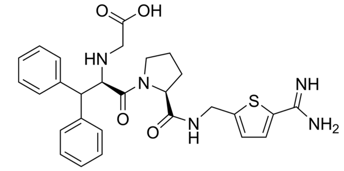
170

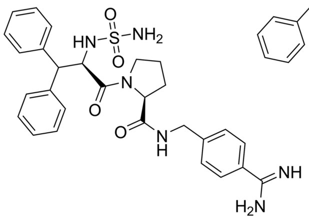
171

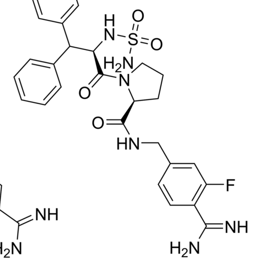
172

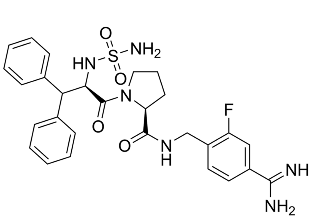
173

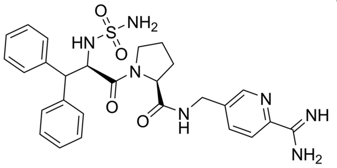
174

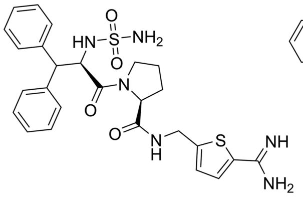
175

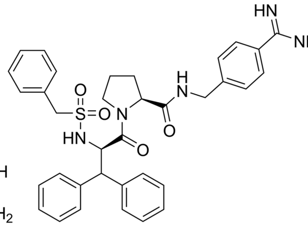
176

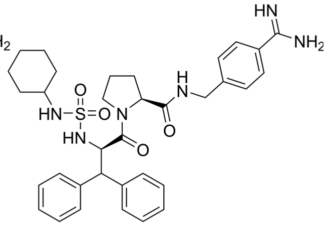
177

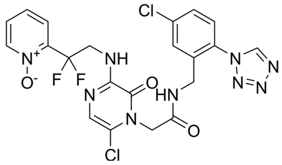
178

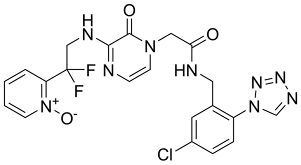
179

Figure S10: Selected actives of thrombin (continued).

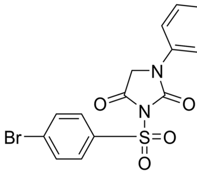

180

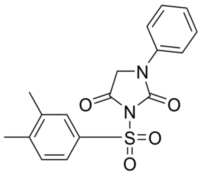

181

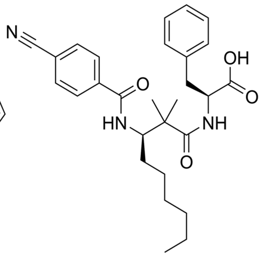

182

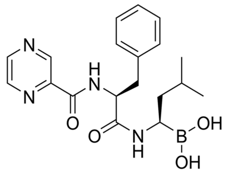

183

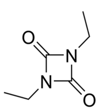

184

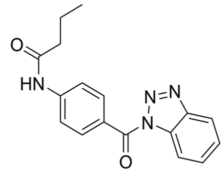

185

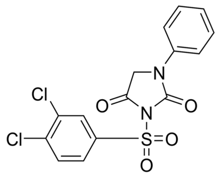

186

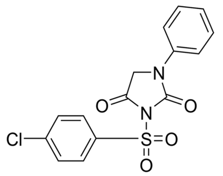

187

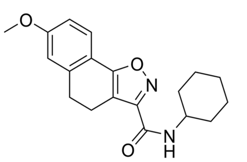

188

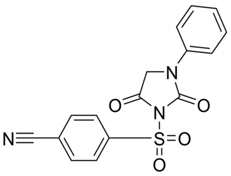

189

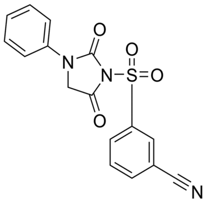

190

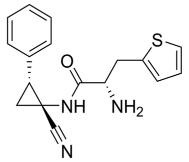

191

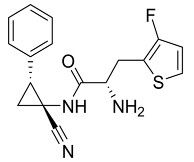

192

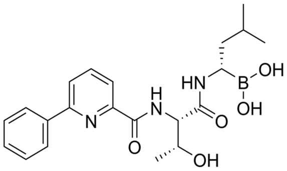

193

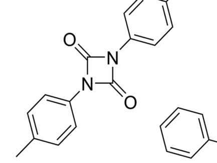

194

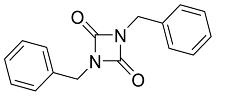

195

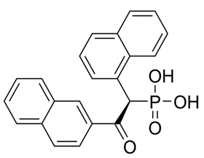

196

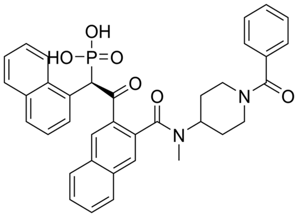

197

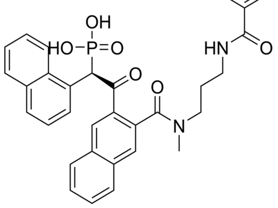

198

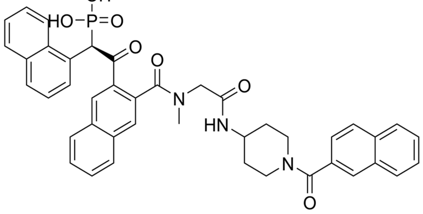

199

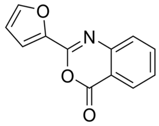

200

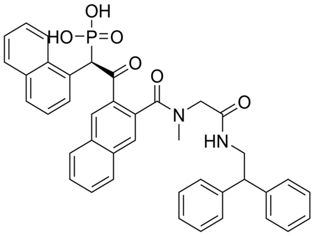

201

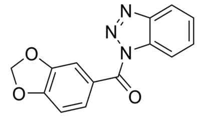

202

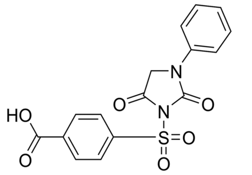

203

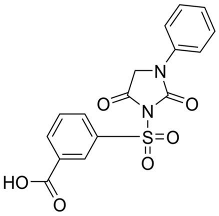

204

Figure S11: Selected actives of cathepsin G.

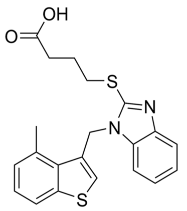

205

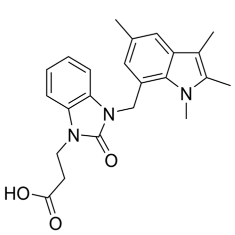

206

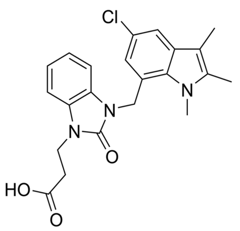

207

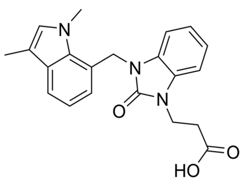

208

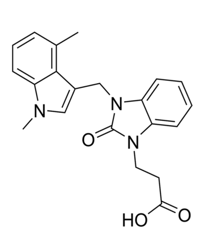

209

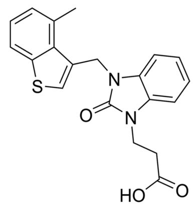

210

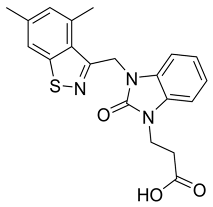

211

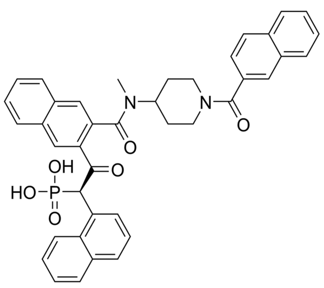

212

Figure S12: Selected actives of cathepsin G (continued).

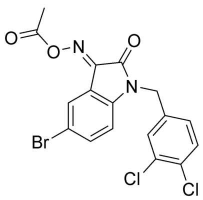

213

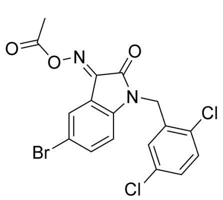

214

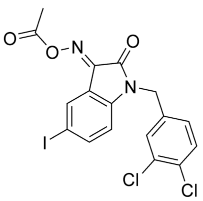

215

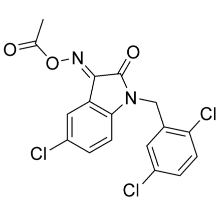

216

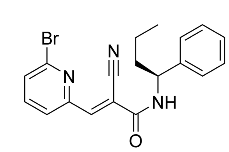

217

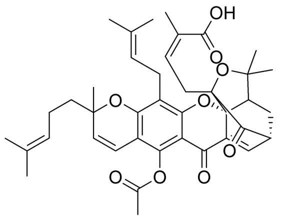

218

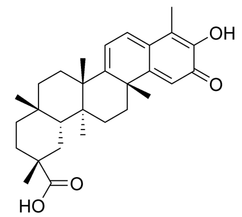

219

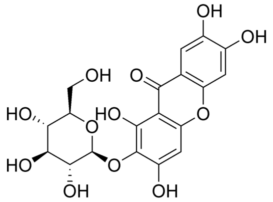

220

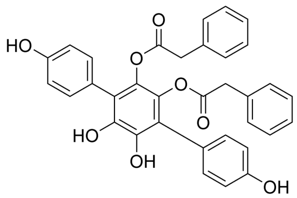

221

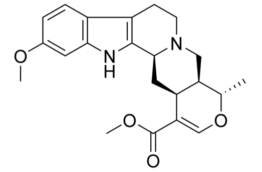

222

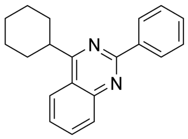

223

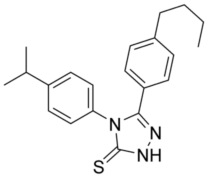

224

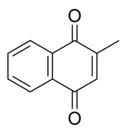

225

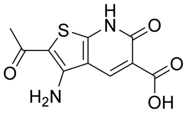

226

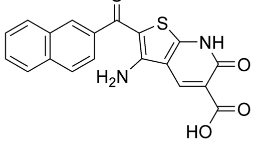

227

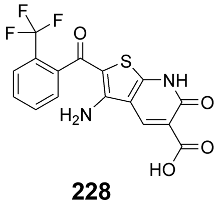

228

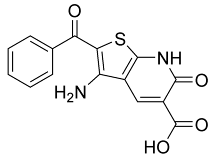

229

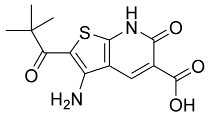

230

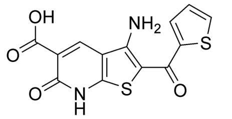

231

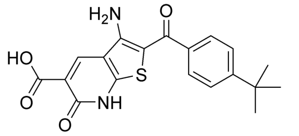

232

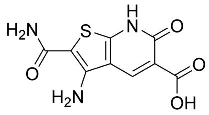

233

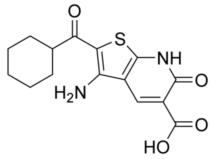

234

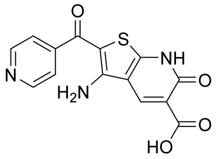

235

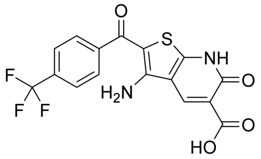

236

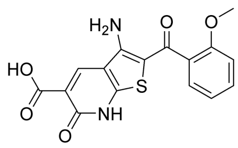

237

Figure S13: Selected actives of UCHL1.

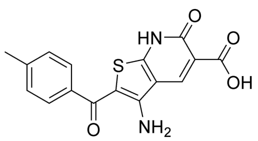

238

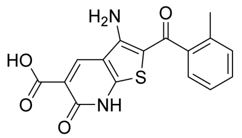

239

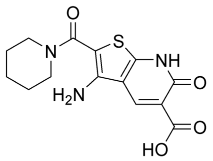

240

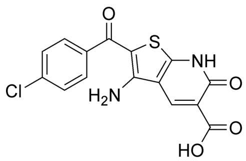

241

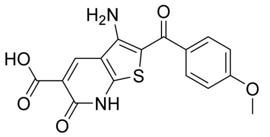

242

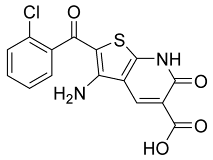

243

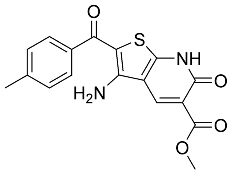

244

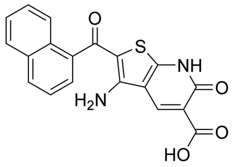

245

Figure S14: Selected actives of UCHL1 (continued).

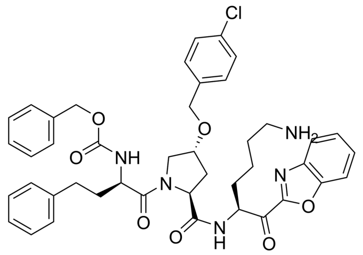

246

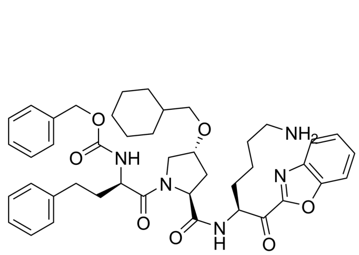

247

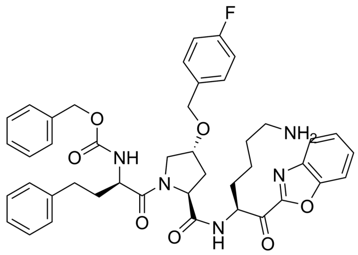

248

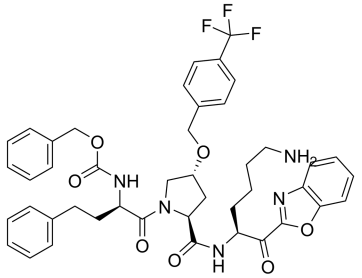

249

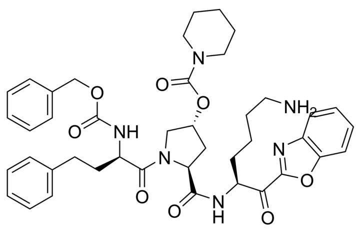

250

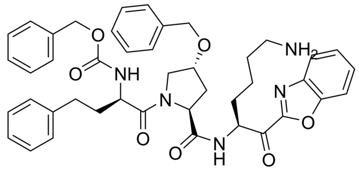

251

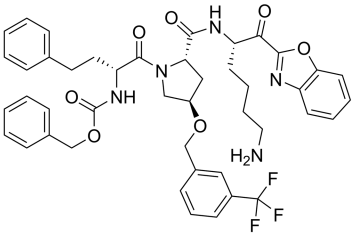

252

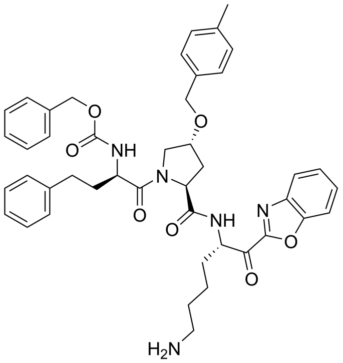

253

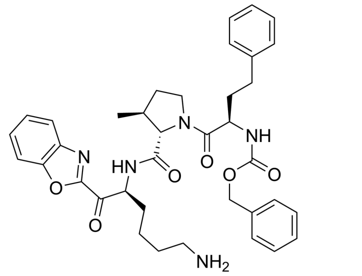

254

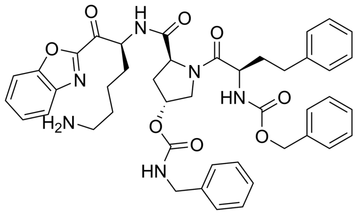

255

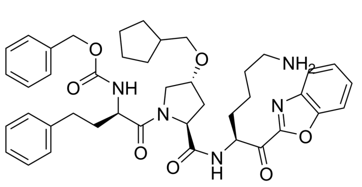

256

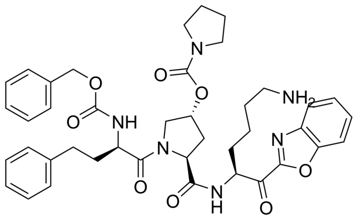

257

Figure S15: Selected actives of prostasin.

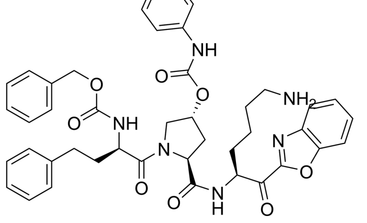

258

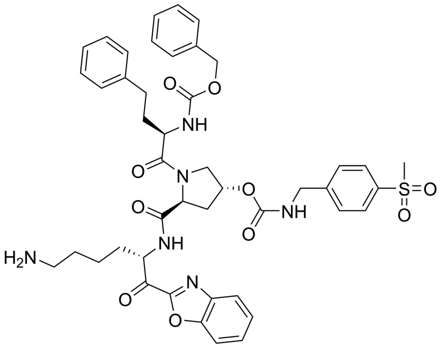

259

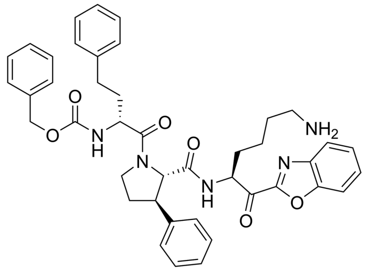

260

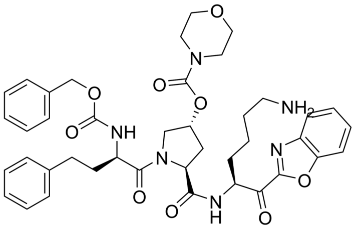

261

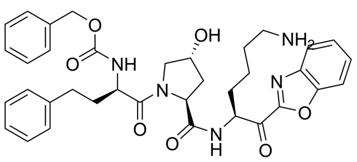

262

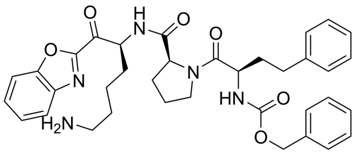

263

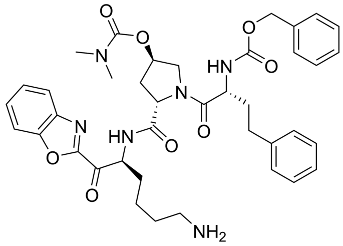

264

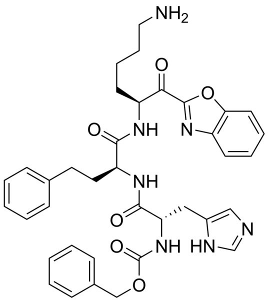

265

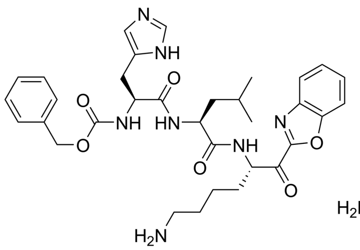

266

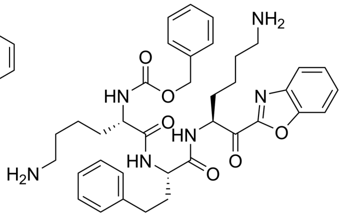

267

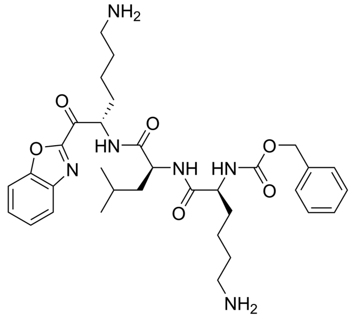

268

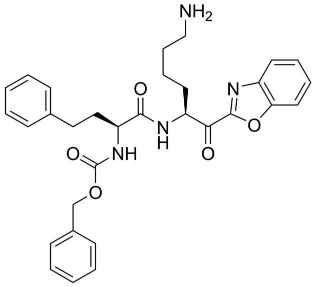

269

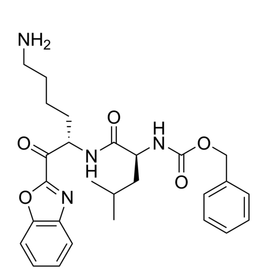

270

Figure S16: Selected actives of prostasin (continued).

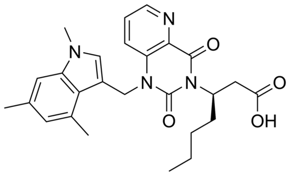
271

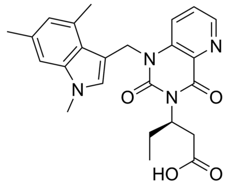
272

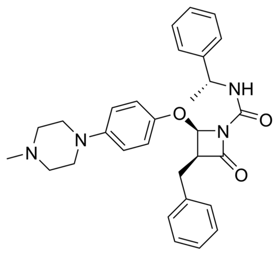
273

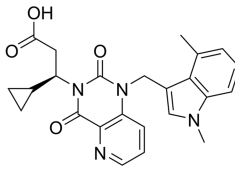
274

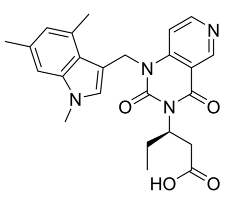
275

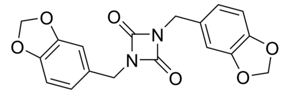
276

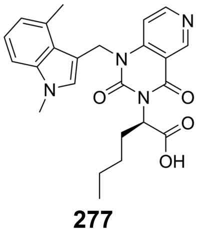
277

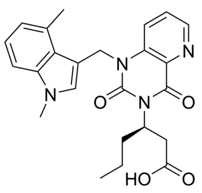
278

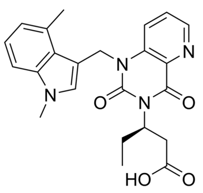
279

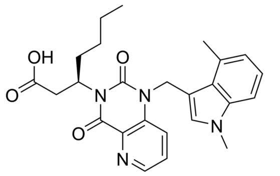
280

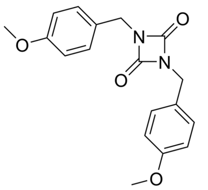
281

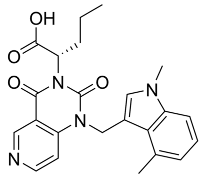
282

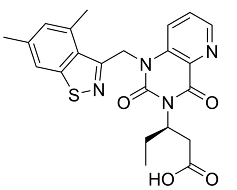
283

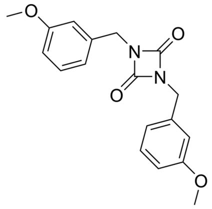
284

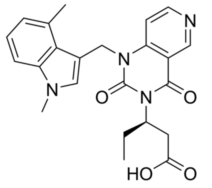
285

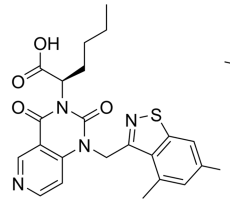
286

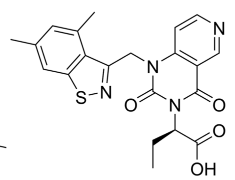
287

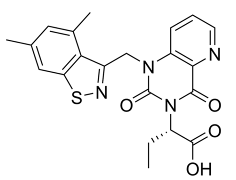
288

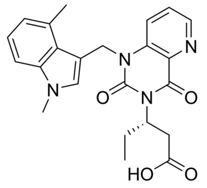
289

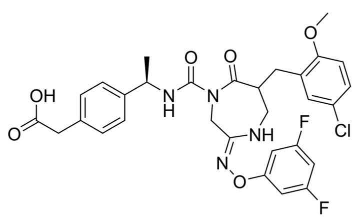
290

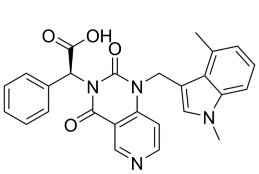
291

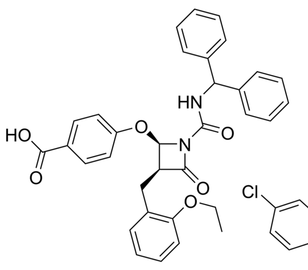
292

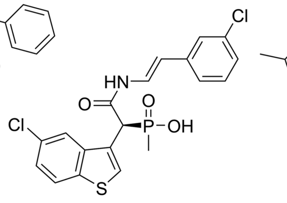
293

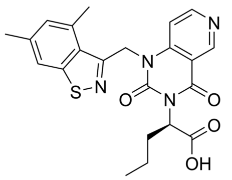
294

Figure S17: Selected actives of chymase.

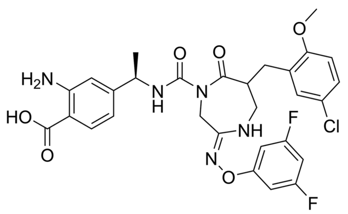

295

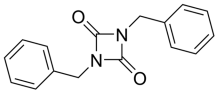

296

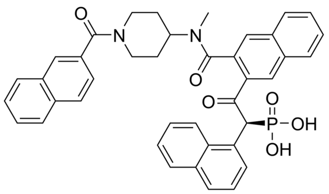

297

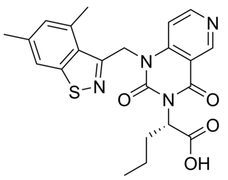

298

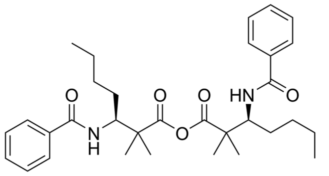

299

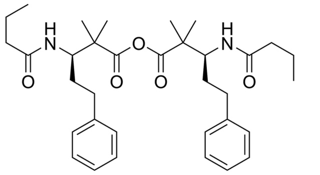

300

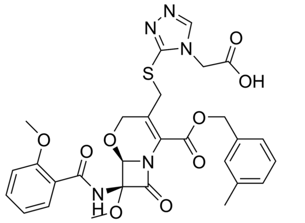

301

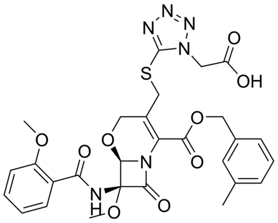

302

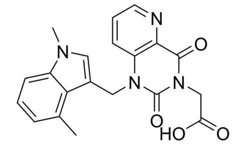

303

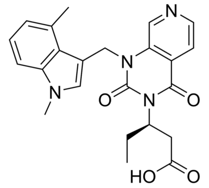

304

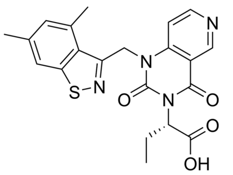

305

Figure S18: Selected actives of chymase (continued).

Table S2: Origin of the SARS-CoV-2 M^{pro} inhibitors.

**Compound**	**Origin**	**Identificator**
Compound 1	Literature	(R)-Beperidil
Compound 2	Literature	(S)-Beperidil
Compound 3	RCSB PDB	5RF7
Compound 4	RCSB PDB	5RFE
Compound 5	RCSB PDB	5RG1
Compound 6	RCSB PDB	5REB
Compound 7	RCSB PDB	5R80
Compound 8	RCSB PDB	5R81
Compound 9	RCSB PDB	5R83
Compound 10	RCSB PDB	5R84
Compound 11	RCSB PDB	5RH3
Compound 12	RCSB PDB	5RF1
Compound 13	RCSB PDB	5R7Y
Compound 14	RCSB PDB	5RGU
Compound 15	RCSB PDB	5RGX
Compound 16	RCSB PDB	5RGI
Compound 17	RCSB PDB	5RGW
Compound 18	RCSB PDB	5RGZ
Compound 19	RCSB PDB	5RGH
Compound 20	RCSB PDB	5RGV
Compound 21	RCSB PDB	5RGY
Compound 22	RCSB PDB	5R7Z
Compound 23	RCSB PDB	5RF6
Compound 24	RCSB PDB	5RH1
Compound 25	RCSB PDB	5RH2
Compound 26	RCSB PDB	5RH8
Compound 27	RCSB PDB	5RH0
Compound 28	RCSB PDB	5REZ
Compound 29	Literature	Ebastine
Compound 30	RCSB PDB	6M2N
Compound 31	Literature	Nelfinavir
Compound 32	Literature	Lopinavir
Compound 33	Literature	Pimozide

Table S3: Origin of the SARS-CoV M^{pro} active compounds.

**Compound**	**Origin**	**Identificator**
Compound 34	Literature	Compound 82
Compound 35	Literature	Compound 83
Compound 36	Literature	Compound 90
Compound 37	Literature	Compound 78
Compound 38	Literature	Compound 77
Compound 39	Literature	Compound 75
Compound 40	Literature	Compound 76
Compound 41	RCSB PDB	3V3M
Compound 42	Literature	Compound 134
Compound 43	Literature	Compound 79
Compound 44	Literature	Compound 80
Compound 45	Literature	Compound 88
Compound 46	Literature	Compound 89
Compound 47	Literature	Compound 86
Compound 48	Literature	Compound 87
Compound 49	Literature	Compound 182
Compound 50	Literature	Compound 81
Compound 51	Literature	Compound 180
Compound 52	Literature	Compound 181
Compound 53	Literature	Compound 189
Compound 54	Literature	Compound 183
Compound 55	Literature	Compound 184
Compound 56	Literature	Compound 133
Compound 57	Literature	Compound 135
Compound 58	Literature	Compound 137
Compound 59	Literature	Compound 136
Compound 60	Literature	Compound 99
Compound 61	Literature	Compound 132
Compound 62	Literature	Compound 129
Compound 63	Literature	Compound 131
Compound 64	Literature	Compound 130
Compound 65	Literature	Compound 140
Compound 66	Literature	Compound 139
Compound 67	Literature	Compound 106
Compound 68	Literature	Compound 104
Compound 69	Literature	Compound 108
Compound 70	Literature	Compound 100
Compound 71	Literature	Compound 110
Compound 72	Literature	Compound 103

Table S4: Origin of the caspase-3 active compounds.

**Compound**	**Origin**	**Identificator**
Compound 73	PubChem BioAssay	Assay 49517
Compound 74	PubChem BioAssay	Assay 49517
Compound 75	PubChem BioAssay	Assay 49517
Compound 76	PubChem BioAssay	Assay 49517
Compound 77	PubChem BioAssay	Assay 49517
Compound 78	PubChem BioAssay	Assay 488901
Compound 79	PubChem BioAssay	Assay 49518
Compound 80	PubChem BioAssay	Assay 49518
Compound 81	PubChem BioAssay	Assay 49518
Compound 82	PubChem BioAssay	Assay 240542
Compound 83	PubChem BioAssay	Assay 240542
Compound 84	PubChem BioAssay	Assay 240542
Compound 85	PubChem BioAssay	Assay 240542
Compound 86	PubChem BioAssay	Assay 240542
Compound 87	PubChem BioAssay	Assay 240542
Compound 88	PubChem BioAssay	Assay 240542
Compound 89	PubChem BioAssay	Assay 240542
Compound 90	PubChem BioAssay	Assay 240542
Compound 91	PubChem BioAssay	Assay 240542
Compound 92	PubChem BioAssay	Assay 241557
Compound 93	Literature	Compound 13
Compound 94	PubChem BioAssay	Assay 241557
Compound 95	Literature	Compound 17
Compound 96	PubChem BioAssay	Assay 241557
Compound 97	PubChem BioAssay	Assay 241557
Compound 98	PubChem BioAssay	Assay 241557
Compound 99	PubChem BioAssay	Assay 240542
Compound 100	PubChem BioAssay	Assay 241557
Compound 101	PubChem BioAssay	Assay 504488
Compound 102	PubChem BioAssay	Assay 588574
Compound 103	PubChem BioAssay	Assay 504488
Compound 104	PubChem BioAssay	Assay 504488
Compound 105	PubChem BioAssay	Assay 588574
Compound 106	PubChem BioAssay	Assay 588574
Compound 107	PubChem BioAssay	Assay 49518
Compound 108	PubChem BioAssay	Assay 49531
Compound 109	Literature	Compound 15
Compound 110	PubChem BioAssay	Assay 504488

Table S5: Origin of the factor Xa active compounds.

Compound	Origin	Identificator
Compound 111	PubChem BioAssay and RCSB PDB	1EZQ
Compound 112	PubChem BioAssay and RCSB PDB	4BTT
Compound 113	PubChem BioAssay and RCSB PDB	3KL6
Compound 114	PubChem BioAssay and RCSB PDB	1MQ5
Compound 115	PubChem BioAssay and RCSB PDB	2P94
Compound 116	PubChem BioAssay and RCSB PDB	4A7I
Compound 117	PubChem BioAssay and RCSB PDB	3M37
Compound 118	PubChem BioAssay and RCSB PDB	3ENS
Compound 119	PubChem BioAssay and RCSB PDB	3LIW
Compound 120	PubChem BioAssay and RCSB PDB	3CEN
Compound 121	PubChem BioAssay and RCSB PDB	2RA0
Compound 122	PubChem BioAssay and RCSB PDB	2PHB
Compound 123	PubChem BioAssay and RCSB PDB	2BQW
Compound 124	PubChem BioAssay and RCSB PDB	1Z6E
Compound 125	PubChem BioAssay and RCSB PDB	3SW2
Compound 126	PubChem BioAssay and RCSB PDB	2CJI
Compound 127	PubChem BioAssay and RCSB PDB	1LPZ
Compound 128	PubChem BioAssay and RCSB PDB	1FJS
Compound 129	PubChem BioAssay and RCSB PDB	1F0R
Compound 130	PubChem BioAssay and RCSB PDB	1WU1
Compound 131	PubChem BioAssay and RCSB PDB	2J34
Compound 132	PubChem BioAssay and RCSB PDB	1G2M
Compound 133	PubChem BioAssay and RCSB PDB	1FAX
Compound 134	PubChem BioAssay and RCSB PDB	2VH0
Compound 135	PubChem BioAssay and RCSB PDB	1G2L
Compound 136	PubChem BioAssay and RCSB PDB	1F0S
Compound 137	PubChem BioAssay and RCSB PDB	2Y82
Compound 138	PubChem BioAssay and RCSB PDB	1V3X
Compound 139	PubChem BioAssay and RCSB PDB	1LPG
Compound 140	PubChem BioAssay and RCSB PDB	2J4I
Compound 141	PubChem BioAssay and RCSB PDB	2P93
Compound 142	PubChem BioAssay and RCSB PDB	2BMG
Compound 143	PubChem BioAssay and RCSB PDB	2JKH
Compound 144	PubChem BioAssay and RCSB PDB	1NFU
Compound 145	PubChem BioAssay and RCSB PDB	1NFW

Table S6: Origin of the cathepsin G active compounds.

Compound	Origin	Identificator
Compound 180	Literature	Compound 6
Compound 181	Literature	Compound 23
Compound 182	Literature	Compound 3
Compound 183	Literature	Bortezomib
Compound 184	Literature	Compound 10
Compound 185	PubChem BioAssay	Assay 832
Compound 186	Literature	Compound 22
Compound 187	Literature	Compound 4
Compound 188	PubChem BioAssay	Assay 832
Compound 189	Literature	Compound 12
Compound 190	Literature	Compound 11
Compound 191	Literature	Compound 1
Compound 192	Literature	Compound 3
Compound 193	Literature	CEP-18770
Compound 194	Literature	Compound 7
Compound 195	Literature	Compound 1
Compound 196	RCSB PDB	1KYN
Compound 197	Literature	Compound 17d
Compound 198	Literature	Compound 17e
Compound 199	Literature	Compound 17f
Compound 200	PubChem BioAssay	Assay 832
Compound 201	Literature	Compound 17c
Compound 202	Pubchem BioAssay	Assay 832
Compound 203	Literature	Compound 17
Compound 204	Literature	Compound 16
Compound 205	Literature	Compound 1
Compound 206	Literature	Compound 42
Compound 207	Literature	Compound 36
Compound 208	Literature	Compound 39
Compound 209	Literature	Compound 31
Compound 210	Literature	Compound 1
Compound 211	Literature	Compound 32
Compound 212	RCSB PDB	1T32

Table S7: Origin of the UCHL1 active compounds.

Compound	Origin	Identificator
Compound 213	Literature	Compound 1
Compound 214	PubChem BioAssay	Assay 506806
Compound 215	Literature	Compound 3
Compound 216	Literature	Compound 2
Compound 217	Literature	WP1130
Compound 218	BindingDB	BDBM50442907
Compound 219	BindingDB	BDBM205457
Compound 220	BindingDB	BDBM50242207
Compound 221	Literature	Vialinin A
Compound 222	BindingDB	BDBM65524
Compound 223	BindingDB	BDBM53421
Compound 224	Literature	Compound 5
Compound 225	Literature	Compound 4
Compound 226	Literature	Compound 29
Compound 227	Literature	Compound 26
Compound 228	Literature	Compound 22
Compound 229	Literature	Compound 7
Compound 230	Literature	Compound 30
Compound 231	Literature	Compound 28
Compound 232	Literature	Compound 18
Compound 233	Literature	Compound 32
Compound 234	Literature	Compound 31
Compound 235	Literature	Compound 27
Compound 236	Literature	Compound 21
Compound 237	Literature	Compound 20
Compound 238	Literature	Compound 16
Compound 239	Literature	Compound 17
Compound 240	Literature	Compound 33
Compound 241	Literature	Compound 23
Compound 242	Literature	Compound 19
Compound 243	Literature	Compound 24
Compound 244	Literature	Compound 9
Compound 245	Literature	Compound 25

Table S8: Origin of the prostasin active compounds.

**Compound**	**Origin**	**Identificator**
Compound 246	Literature	Compound 23
Compound 247	Literature	Compound 24
Compound 248	Literature	Compound 22
Compound 249	Literature	Compound 20
Compound 250	Literature	Compound 29
Compound 251	Literature	Compound 17
Compound 252	Literature	Compound 19
Compound 253	Literature	Compound 21
Compound 254	Literature	Compound 16
Compound 255	Literature	Compound 27
Compound 256	Literature	Compound 25
Compound 257	Literature	Compound 30
Compound 258	Literature	Compound 26
Compound 259	Literature	Compound 28
Compound 260	Literature	Compound 15
Compound 261	Literature	Compound 31
Compound 262	Literature	Compound 18
Compound 263	Literature	Compound 14
Compound 264	Literature	Compound 32
Compound 265	Literature	Compound 13
Compound 266	Literature	Compound 11
Compound 267	Literature	Compound 12
Compound 268	Literature	Compound 10
Compound 269	Literature	Compound 9
Compound 270	Literature	Compound 8

Table S9: Origin of the thrombin active compounds.

**Compound**	**Origin**	**Identificator**
Compound 146	Literature	Compound 17
Compound 147	Literature	Compound 24
Compound 148	Literature	Compound 40
Compound 149	Literature	Compound 2
Compound 150	Literature	Compound 20
Compound 151	Literature	Compound 5
Compound 152	Literature	Compound 16
Compound 153	Literature	Compound 13
Compound 154	Literature	Compound 3
Compound 155	Literature	Compound 16
Compound 156	Literature	Compound 8
Compound 157	Literature	Compound 9a
Compound 158	Literature	Compound 18
Compound 159	Literature	Compound 16
Compound 160	Literature	Compound 17
Compound 161	Literature	Compound 13
Compound 162	Literature	Compound 14
Compound 163	Literature	Compound 17
Compound 164	Literature	Compound 6
Compound 165	Literature	Compound 18
Compound 166	Literature	Compound 33
Compound 167	Literature	Compound 5
Compound 168	Literature	Compound 25
Compound 169	Literature	Compound 11n
Compound 170	Literature	Compound 22
Compound 171	Literature	Compound 11f
Compound 172	Literature	Compound 4
Compound 173	Literature	Compound 5
Compound 174	Literature	Compound 6
Compound 175	Literature	Compound 7
Compound 176	Literature	Compound 11g
Compound 177	Literature	Compound 11h
Compound 178	Literature	Compound 34
Compound 179	Literature	Compound 38

Table S10: Origin of the chymase active compounds.

**Compound**	**Origin**	**Identificator**
Compound 271	US Patent	US8501749
Compound 272	US Patent	US8501749
Compound 273	PubChem BioAssay	Assay 547676
Compound 274	US Patent	US8501749
Compound 275	US Patent	US8501749
Compound 276	Literature	Compound 6
Compound 277	US Patent	US8501749
Compound 278	US Patent	US8501749
Compound 279	US Patent	US8501749
Compound 280	US Patent	US8501749
Compound 281	Literature	Compound 4
Compound 282	US Patent	US8501749
Compound 283	US Patent	US8501749
Compound 284	Literature	Compound 5
Compound 285	US Patent	US8501749
Compound 286	US Patent	US8501749
Compound 287	US Patent	US8501749
Compound 288	US Patent	US8501749
Compound 289	US Patent	US8501749
Compound 290	US Patent	US8501749
Compound 291	US Patent	US8501749
Compound 292	Literature	(3S,4S)-4-(4-Carboxy)phenoxy-1-[diphenylmethylaminocarbonyl]-3-(2-ethoxybenzyl)azetidin-2-one
Compound 293	Literature	Compound 6g
Compound 294	US Patent	US8501749
Compound 295	US Patent	US8501749
Compound 296	Literature	Compound 3
Compound 297	Literature	Compound 1
Compound 298	US Patent	US8501749
Compound 299	Literature	Compound 12
Compound 300	Literature	Compound 15
Compound 301	BindingDB	BDBM50093722
Compound 302	Literature	Compound 1
Compound 303	US Patent	US8501749
Compound 304	US Patent	US8501749
Compound 305	US Patent	US8501749

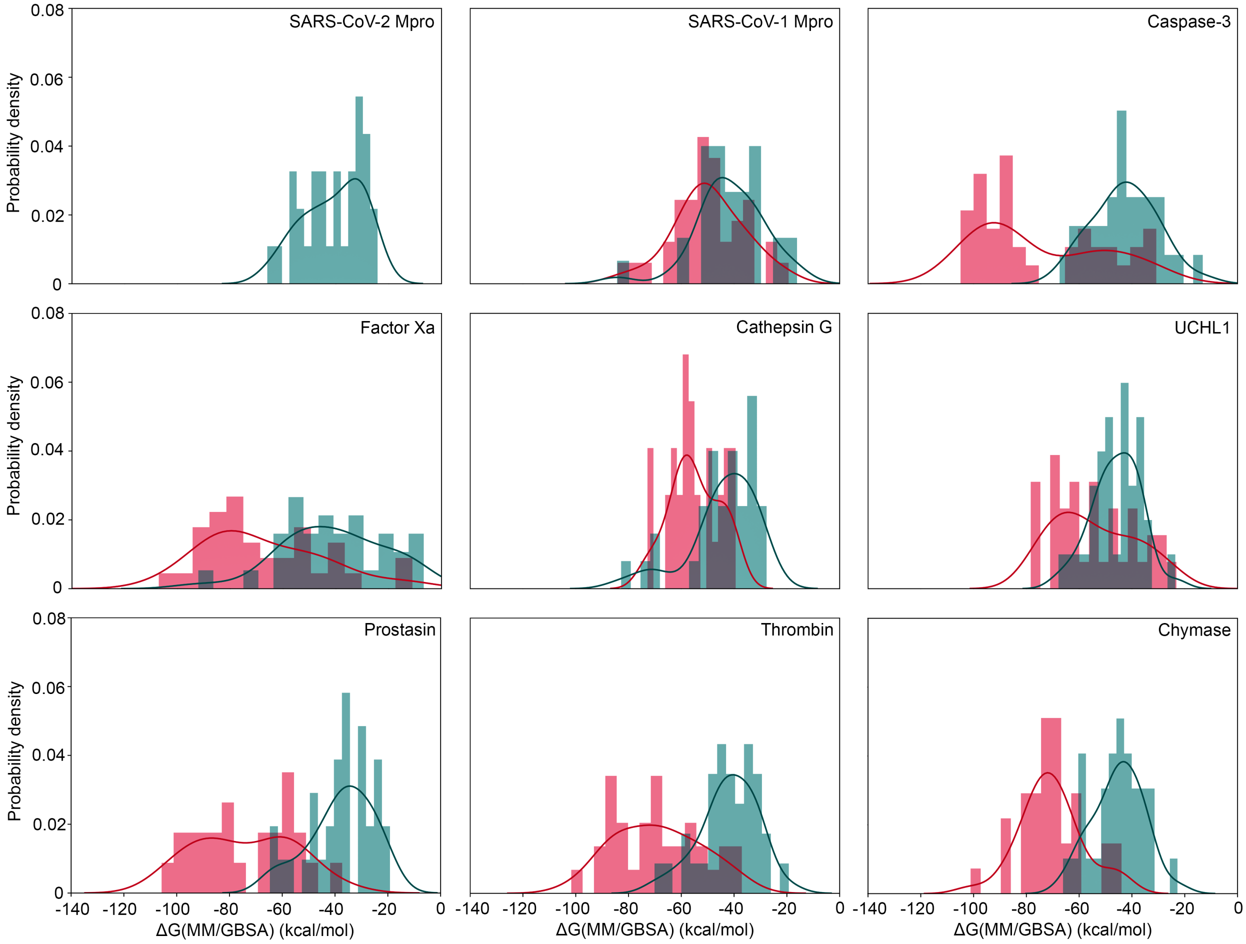

Figure S19: Binding free energies obtained with the MM/GBSA procotol from post-processing docking poses (of SARS-CoV-2 M^{pro} inhibitors docked to the selected panel of proteases) with MD simulations. The compounds designed against SARS-CoV-2 M^{pro} are shown in pine green, while the known actives for the remaining targets are shown in red.

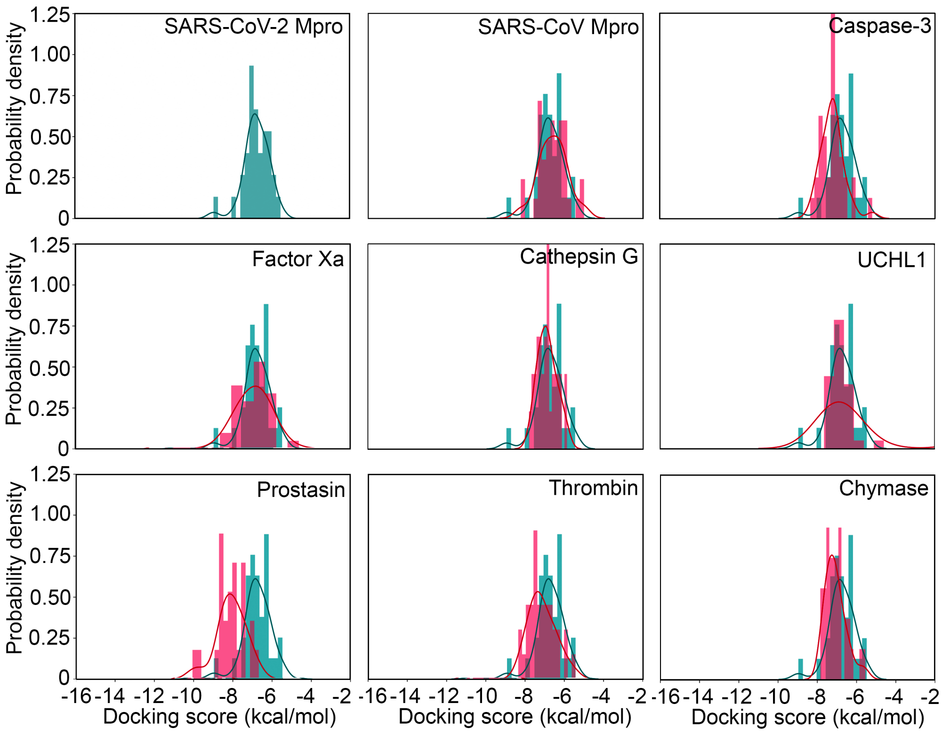

Figure S20: Distribution of actives of the respective target to SARS-CoV-2 M^{pro}. The native SARS-CoV-2 M^{pro} inhibitors are shown in pine green, while the other compounds are colored red.

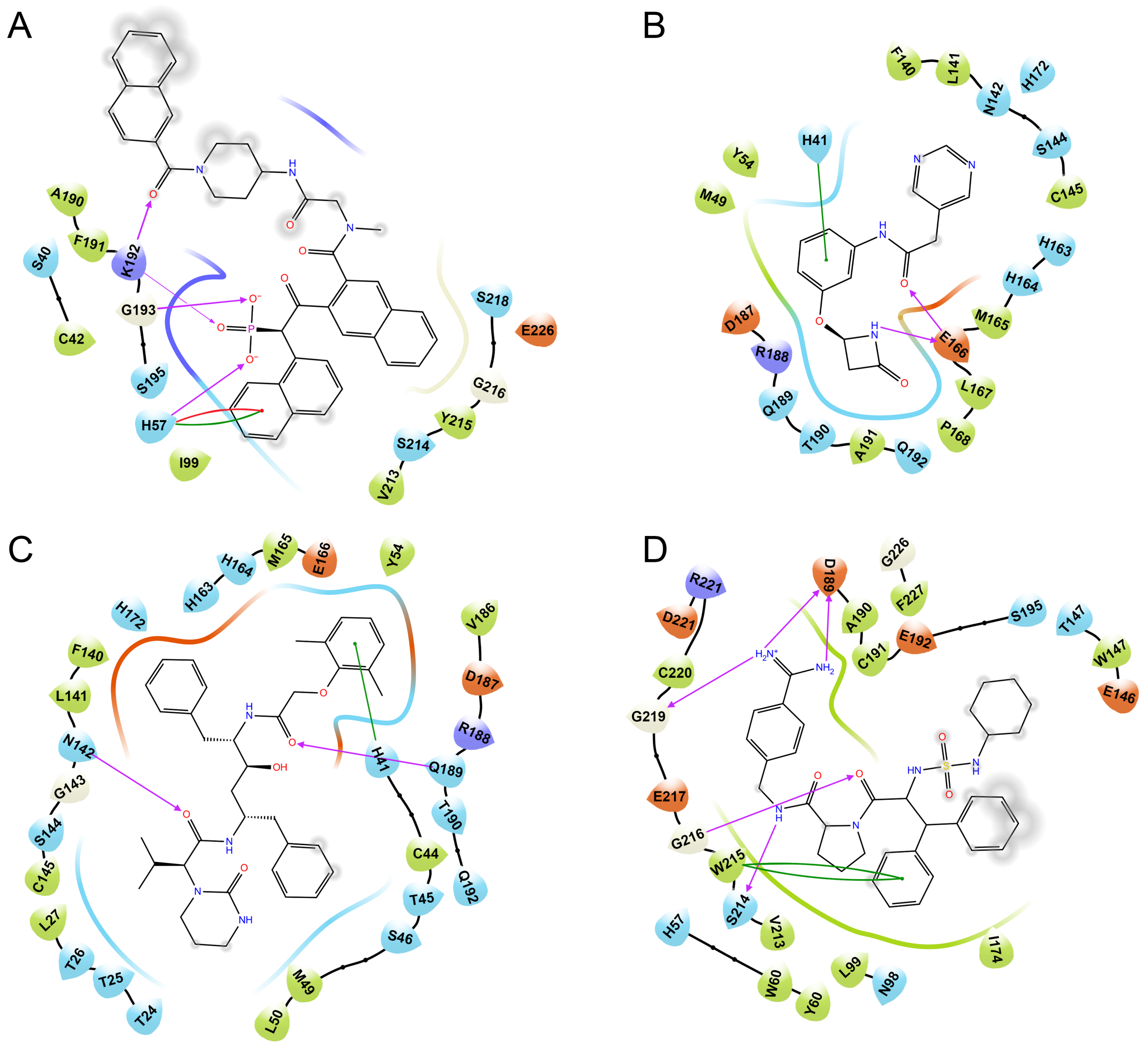

Figure S21: 2D depiction of binding modes of (A) compound **199** toward cathepsin G, (B) compound **14** toward SARS-CoV-2 Mpro, (C) compound **32** toward SARS-CoV-2 Mpro, and (D) compound **177** toward thrombin.

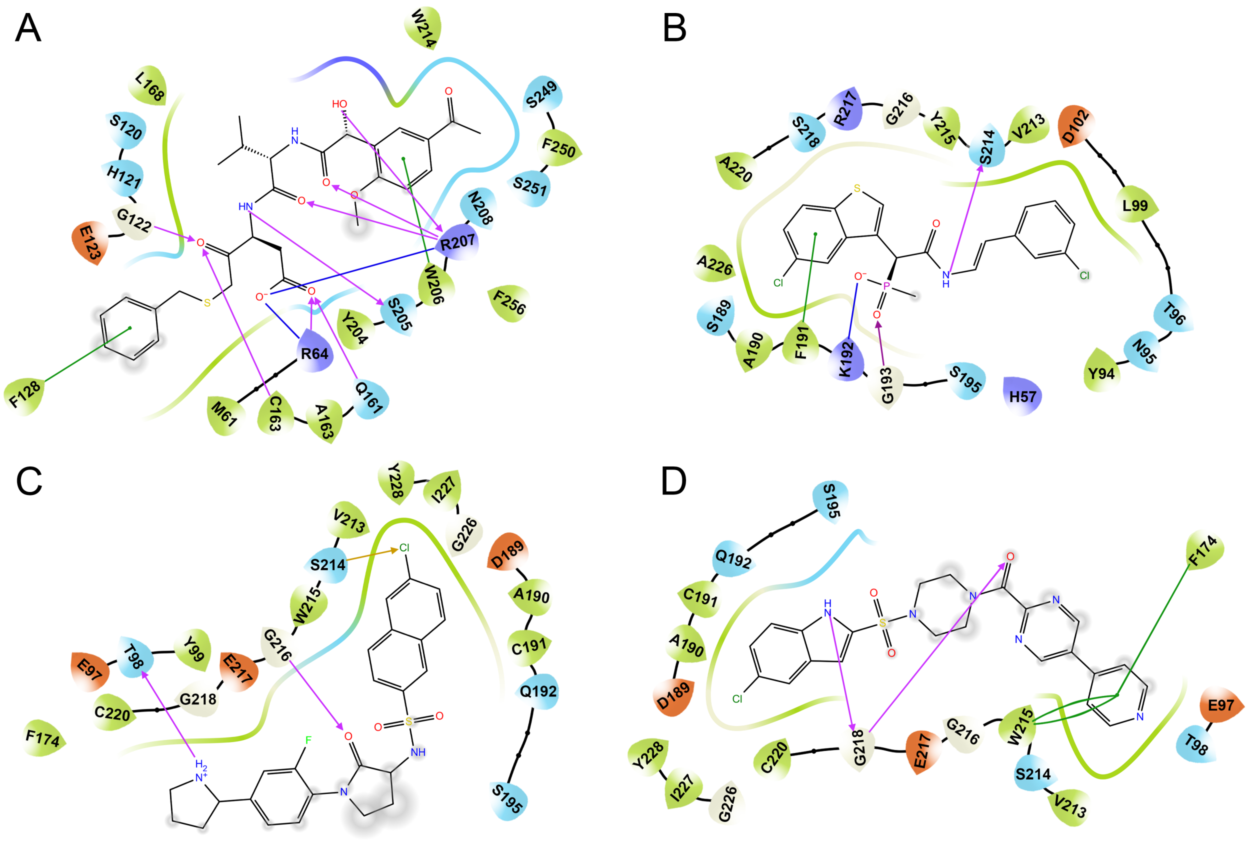

Figure S22: 2D depiction of binding modes of (A) compound **86** toward caspase-3, (B) compound **293** toward chymase, (C) compound **137** toward factor Xa, and (D) compound **130** toward factor Xa.

Table S11: Structures used to assess each target's cross-docking performance.

Protein	Ligands	X-ray structures	Ensemble size	Percentage[a]	Method
SARS-CoV-2 M^{pro}	27	41	8	96.3%	Glide SP
SARS-CoV M^{pro}	7	17	6	85.7%	Smina
Caspase-3	4	11	1	75.0%	Glide SP
Factor Xa	126	128	5	96.8%	Smina
Cathepsin G	4	4	1	100.0%	Smina
UCHL1	0	7	7	n/a [b]	Smina
Prostasin	2	9	1	50.0%	Smina
Thrombin	59	59	3	98.3%	Smina
Chymase	8	12	2	87.5%	Glide SP

[a] The percentage of docked poses with an RMSD below 2.5 Å to the co-crystallized pose.
[b] Percentage could not be calculated due to a lack of co-crystallized ligands.

Table S12: Structures and metrics used to validate the performance of the docking.

**Protein**	**Actives**	**Decoys**	**ROC AUC**	**Enrichment factor**
SARS-CoV-2 M^{pro}	34	1360	0.631	40.71
SARS-CoV M^{pro}	39	1000	0.911	26.64
Caspase-3	38	400	0.863	11.53
Factor Xa	35	650	0.953	19.57
Cathepsin G	33	750	0.864	23.06
UCHL1	7	100	0.810	15.29
Prostasin[a]	16	n/a	n/a	n/a
Thrombin[a]	34	n/a	n/a	n/a
Chymase	35	350	0.899	10.91

No performance metrics were calculated because no decoys could be generated using the DUDE-Z server.

Toxicity profiling

Table S13: The toxic potential for all tested SARS-CoV-2 actives as predicted by Virtual-ToxLab.

Compound	Toxic potential
1	0.541
2	0.522
3	0.202
4	0.255
5	0.485
6	0.365
7	0.423
8	0.458
9	0.398
10	0.376
11	0.296
12	0.398
13	0.364
14	0.249
15	0.194
16	0.290
17	0.130
18	0.213
19	0.307
20	0.501
21	0.239
22	0.288
23	0.305
24	0.394
25	0.308
26	0.332
27	0.234
28	0.262
29	0.578
30	0.488
31	0.517
32	0.535
33	0.383

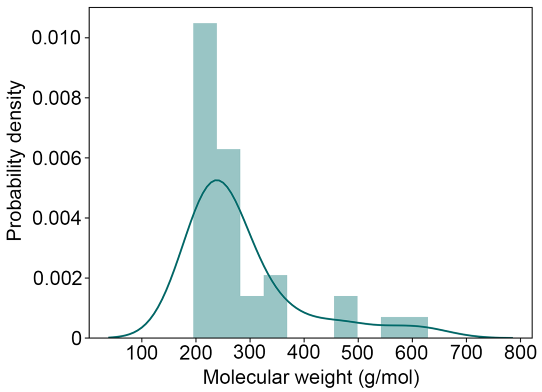

Figure S23: Distribution of the molecular weight of the tested SARS-CoV-2 actives.

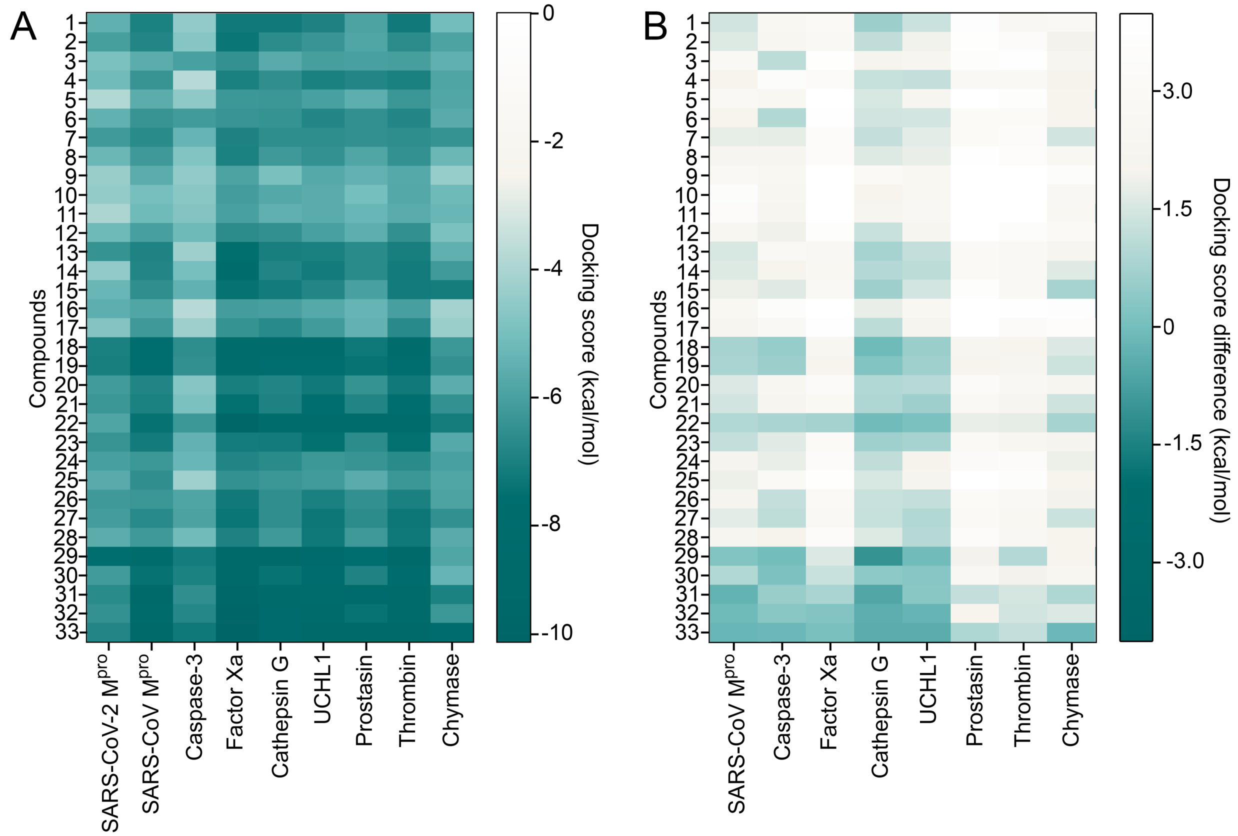

Figure S24: Comparison of docking scores. To obtain absolute values, compounds docked with Glide SP were rescored with smina. (A) Docking scores of compounds **1-33** to the selected panel of proteases. (B) Difference of the docking scores of SARS-CoV-2 M^{pro} inhibitors and the average score of actives binding to the respective target. Negative values indicate binding of the SARS-CoV-2 M^{pro} inhibitors to the off-target.

Supporting Materials and Methods

Model preparation

The crystal structures used for docking as well as the validation of the docking protocols (Tables S14-16) were prepared using the Protein Preparation Wizard[64] by adding hydrogen atoms, assigning bond orders, assessing protonation states at pH 7.4, and orienting the hydrogen bonding network followed by a restrained minimization. We conducted the minimization with a convergence threshold of 0.3 Å for the protein heavy atoms using the OPLS3e force field. Crystallographic water molecules were discarded and the cocrystallized ligands were obtained from the prepared structures. The remaining ligands were treated with the LigPrep protocol in the Maestro Small-Molecule Drug Discovery Suite[65] with Epik to predict the protonation states at pH 7.4 and the OPLS3 force field to obtain low-energy conformers. For MD simulations, crystal structures were retrieved from the Protein Data Bank[66] (Table S15). In the case of caspase-3 and factor Xa structures, the cocrystallized ligands were removed manually from the crystal structures.

MAV calculation

AQUA-DUCT 1.0 software was also used for the calculation of the maximum available volume (MAV) of the active site of each selected protease. The MAV value correspond to the volume of the outer pocket calculated by the *pond* module. *Pond* module calculates pockets prior to the hot-spots identification to determine the space of a particular macromolecule which was visited by traced molecules (here: water molecules) during the simulation time. There are two types of pockets: inner pocket represents places that were very often visited, while the outer pocket stands for all the regions that were penetrated. For the MAV value calculation only the information on those water molecule that entered and/or left the active site of a particular protease was used, therefore it represents the volume of the active site space which was penetrated by water molecules during the simulation time.

Table S14: Name and UniProt IDs of the proteases used in this work.

**Protein**	**UniProt ID**
SARS-CoV-2 M^{pro}	P0DTD1
SARS-CoV M^{pro}	P0C6X7
Caspase-3	P42574
Factor Xa	P00742
Cathepsin G	P08311
UCHL1	P09936
Prostasin	Q16651
Thrombin	P00734
Chymase	P23946

Table S15: Number of water molecules and counter ions used for the MD simulations.

**Protein**	**PDB ID**	**Water molecules**^{a}	**Ions**^{b}
SARS-CoV-2 M^{pro}	6Y2E	22388	4 Na^{+}
SARS-CoV M^{pro}	1Q2W	22880	3 Na^{+}
Caspase-3	2DKO	21531	1 Cl^{-}
Factor Xa	3KL6	16610	none
Cathepsin G	6VTM	10483	24 Cl^{-}
UCHL1	2ETL	9977	5 Na^{+}
Prostasin	3DFJ	13456	6 Na^{+}
Thrombin	3VXE	10529	5 Cl^{-}
Chymase	4AFQ	11264	14 Cl^{-}

^{a} Number of added water molecules during system preparation. ^{b} Number of added ions during system preparation.

Table S16: Crystal structures considered for each target.

Protein	Crystal structures	MD ensemble[a]
SARS-CoV-2 M^{pro}	5R7Y, 5R7Z, 5R8T, 5R80, 5R81, 5R82, 5R83, 5R84, 5RE4, 5REB, 5REZ, 5RF1, 5RF3, 5RF, 5RF7, 5RFE, 5RG1, 5RGH, 5RGI, 5RGU, 5RGV, 5RGW, 5RGX, 5RGY, 5RGZ, 5RH0, 5RH1, 5RH2, 5RH3, 5RH8, 6LU7, 6LZE, 6M0K, 6M2N, 6M2Q, 6W63, 6Y2E, 6YB7, 7BQY, 7BRO, 7BUY	6LU7
SARS-CoV M^{pro}	1UJ1, 1UK3, 2GZ7, 2GZ8, 2H2Z, 2OP9, 3D62, 3EA7, 3F9G, 3SN8, 3SNA, 3TIU, 3V3M, 4MDS, 4TWW, 4WY3, 5N19	2GZ8
Caspase-3	1QX3, 3GJQ, 3GJR, 3GJT, 4EHA, 4EHL, 4QUD, 4QUL, 5I9T, 5IAB, 5IAE	4QUL
Factor Xa	1IQM, 2Y82, 2VH0, 2XC0, 2XBV, 1G2M, 2Y80, 2EI8, 1F0S, 2VWN, 1F0R, 2P16, 2BOH, 2UWP, 2CJI, 2BMG, 1IQG, 2P3T, 2WYJ, 2WYG, 4Y7A, 1IQK, 1Z6E, 2XBY, 1NFU, 1NFY, 2W26, 3ENS, 3LIW, 1XKA, 2VVC, 1FJS, 1LPG, 2BQW, 1LPK, 2EI6, 3CEN, 1IQH, 1LQD, 2Y81, 2VH6, 2P94, 1IQN, 4BTU, 3TK5, 2G00, 2UWO, 2XC5, 1IQF, 2BOK, 3M36, 2P3U, 4Y76, 4A7I, 3KQE, 2P93, 1KSN, 3KQD, 3K9X, 1IOE, 2VWO, 2J34, 2EI7, 4Y79, 3KQC, 2D1J, 1V3X, 2W3K, 2VVU, 1G2L, 2UWL, 2Q1J, 2XBW, 2BQ7, 2J38, 2J95, 1MQ5, 2J4I, 2Y5F, 2Y5H, 2VWL, 2Y5G, 3Q3K, 4ZHA, 4BTT, 1IQJ, 1XKB, 3TK6, 3FFG, 1IQL, 1MQ6, 1IQI, 3HPT, 2PR3, 2XC4, 4Y7B, 5K0H, 3KL6, 1EZQ, 4Y6D, 2VWM, 4BTI, 4Y71, 2VVV, 1IQE, 2Y7X, 2J94, 4ZH8, 2Y7Z, 1NFX, 2J2U, 2PHB, 2JKH, 3KQB, 2BQ6, 1FAX, 1WU1, 3SW2, 3IIT, 1NFW, 2XBX, 2FZZ, 3M37, 1LPZ, 3CS7, 2P95, 2RA0, 2W3I	none
Cathepsin G	1AU8, 1CGH, 1KYN, 1T32	none
Prostasin	3DFJ, 3DFL, 3E0N, 3E0P, 3E16, 3E1X, 3FVF, 3GYL, 3GYM	none

[a] PDB ID of the structure used for ensemble generation using MD simulations.

Table S17: Crystal structures considered for each target (continued).

Protein	Crystal structures	MD ensemble[a]
UCHL1	2ETL, 3IFW, 3IRT, 3KVF, 3KW5, 4DM9, 4JKJ	none
Thrombin	3LDX, 3P17, 3QTO, 3QTV, 3QWC, 3QX5, 3RLW, 3RLY, 3RM0, 3RM2, 3RML, 3RMM, 3RMN, 3RMO, 3SHA, 3SHC, 3SI3, 3SI4, 4SV2, 3T5F, 3TU7, 3U98, 3U9A, 3UTU, 3UWJ, 4AX9, 4BAH, 4BAK, 4BAM, 4BAN, 4BAO, 4BAQ, 4HFP, 4LOY, 4LXB, 4UDW, 4UFD, 4UFE, 4UFF, 4UFG, 4YES, 5A2M, 5AF9, 5AFZ, 5JFD, 5JZY, 5LCE, 5LPD, 5MJT, 5MLS, 5MM6, 6EO9, 6GBW, 6GWE, 6HSX, 6ROT, 6T3Q, 69T4A, 6TDT	none
Chymase	1NN6, 1T31, 2HVX, 3N7O, 3S0N, 4K2Y, 4K5Z, 4K60, 4K69, 4KP0, 5YJM, 5YJP	none

[a] PDB ID of the structure used for ensemble generation using MD simulations.

Table S18: PDB IDs of the proteases which were used in molecular docking.

Protein	Docking
SARS-CoV-2 M^{pro}	5R8T, 5R81, 5REZ, 5RF7, 5RGI, 5RH3, 6LU7, 7BUY
SARS-CoV M^{pro}	2GZ7, 2OP9, 3V3M, 4MDS, 4TWW, 4WY3
Caspase-3	4QUL
Factor Xa	1FJS, 1LPK, 1IQH, 1LQD, 3SW2
Cathepsin G	1T32
UCHL1	2ETL, 3IFW, 3IRT, 3KVF, 3KW5, 4DM0, 4JKJ
Prostasin	3GYM
Thrombin	3TU7, 4LXB, 6GBW
Chymase	2HVX, 5YJP

References

64. Madhavi Sastry, G.; Adzhigirey, M.; Day, T.; Annabhimoju, R.; Sherman, W. Protein and ligand preparation: Parameters, protocols, and influence on virtual screening enrichments. *J. Comput.-Aided Mol. Des.* **2013**, *27*, 221–234, doi:10.1007/s10822-013-9644-8.

65. Schrödinger, LCC, S. *Maestro Small-Molecule Drug Discovery Suite 2019-3*; New York, NY, USA 2019.

66. Berman, H. M.; Westbrook, J.; Feng, Z.; Gilliland, G.; Bhat, T. N.; Weissig, H.; Shindyalov, I. N.; Bourne, P. E. The Protein Data Bank. *Nucleic Acids Res.* **2000**, *28*, 235–242, doi:10.1093/nar/28.1.235.
